# Investigating the molecular transmission dynamics of *bla*_NDM_ in antibiotic-selective environments

**DOI:** 10.1128/jb.00133-25

**Published:** 2025-08-11

**Authors:** Shashi Kumari, Lekshmi Narendrakumar, Meenal Chawla, Sanjib Das, Hemanta Koley, Bhabatosh Das

**Affiliations:** 1 Microbial Research Centre, BRIC-Translational Health Science and Technology Institute 145787, Faridabad, Haryana, India; 2 Division of Bacteriology, ICMR- National Institute for Research in Bacterial Infections 30170 , Kolkata, West Bengal, India; University of Notre Dame, Notre Dame, Indiana, USA

**Keywords:** antibiotic resistance, horizontal gene transfer, mobile genetic elements, insertion sequences, metallo-beta-lactamases

## Abstract

Carbapenem resistance mediated by *bla*_NDM_-encoded metallo-beta-lactamases is often linked to IS*Aba125*, an insertion sequence from the IS*30* family, which is widely distributed among critical and high-priority bacterial pathogens. The rapid dissemination of IS*Aba125*-linked *bla*_NDM_ in both nosocomial and community-acquired infections presents a serious challenge to healthcare systems and pharmaceutical industries. Despite the urgency of this issue, the factors driving *bla*_NDM_ spread and the molecular mechanisms governing IS*Aba125* mobility remain poorly understood. In this study, we engineered the genomes of *Vibrio cholerae* and *Escherichia coli* to investigate the mobility of *bla*_NDM_ under controlled conditions both with and without the genetically linked IS*Aba125*. We also examined the transmission efficiency and the stability of *bla*_NDM_ in environments with and without sublethal antibiotic concentrations. Our *in vitro* findings were validated in a rabbit ileal loop model. The results revealed that antibiotic pressure significantly influences the mobility of *bla*_NDM_, shedding light on the molecular dynamics of its transmission. These insights are crucial for developing strategies to curb the spread of *bla*_NDM_ and mitigate the growing threat of carbapenem resistance in bacterial pathogens.

**IMPORTANCE:**

Insertion sequences are the simplest form of mobile genetic elements that play a critical role in the adaptation of bacteria, allowing them to rapidly acquire new traits like resistance genes that enhance their survival. IS*Aba125* is one such insertion sequence that facilitates the spread of *bla*_NDM_, contributing to the global challenge of carbapenem resistance. In this study, we developed reporter strains that could be used as a valuable tool for investigating the dynamics of IS*Aba125-*linked *bla*_NDM_*sh-ble* and evaluated the transposition frequency of IS*Aba125-*linked *bla*_NDM_*sh-ble* in the presence and absence of sublethal concentration of antibiotics. Our results demonstrated that IS*Aba125* enhances the spread of *bla*_NDM_*sh-ble* under sublethal concentration of antibiotics that induces SOS response.

## INTRODUCTION

Antimicrobial resistance (AMR) poses a growing and urgent threat to both human and animal health, with the recent rise of bacterial pathogens that produce carbapenemases further complicating treatment options. Carbapenems, being one of the last-resort antibiotics, are crucial in combating severe hospital-acquired infections, making this emergence particularly concerning (1). The New Delhi metallo-beta-lactamases (NDM), which fall under the Amber Class B beta-lactamase, are the most widely distributed carbapenemases in enteric pathogens conferring resistance to nearly all beta-lactam antibiotics, except aztreonam. Previously, we demonstrated the difference in genetic arrangements between *bla*_NDM-1_ and *bla*_NDM-5_ across different organisms and compared the different mobile genetic elements (MGEs) associated with these genes. Our findings revealed the association of *bla*_NDM_ genes to IS*30*-like IS*Aba125* or IS*91*-like IS*VSa3* insertion sequences (2). Insertion sequences (IS) are small, highly recombining, and widely distributed transposable elements that are among the most frequent MGEs, facilitating genetic exchange between pathogens and enhancing their adaptability to diverse environments (3). These elements are integrated into chromosomes as well as on plasmids, underscoring their critical role in the dissemination of associated genes. The IS*Aba125* element commonly associated with the Tn125 transposon typically contains terminal inverted repeats (TIRs) at both ends, encoding integrase, recombinase, and transposase enzymes crucial for chromosomal integration and gene transfer (4). Moreover, it has been reported that a decanucleotide, 5′-GAGATAATTG-3′ in the right end of the IS*Aba125* insertion sequence enhances the transposition activity in *Escherichia coli* and exhibits a preference for “natural” hot spots characterized by a 24 bp symmetric consensus in the bacterial genome (5, 6). Additionally, several other insertion sequences in close vicinity of the IS*Aba125* are known to influence its transposition efficacy and gene expression (7). Thus, among the MGEs implicated in *bla*_NDM_ dissemination, IS*Aba125* has emerged as a particularly influential element.

Clinically significant antibiotics can directly and indirectly influence the transmissibility and stability of resistance genes in bacteria (8). Previous studies have shown that sublethal antibiotic concentrations in the environment act as a major driver of resistance development, promoting mutations that enhance bacterial fitness (9, 10). However, the mechanisms through which sublethal antibiotic concentrations promote horizontal gene transfer (HGT) remain poorly understood. Understanding the impact of sublethal antibiotic exposure on MGEs and their transmission dynamics is important for developing effective strategies to combat the spread of AMR. This study aims to investigate how the MGE IS*Aba125* influences the propagation of *bla*_NDM_ in bacterial communities exposed to both normal and sublethal antibiotic concentrations, emphasizing the urgent need for responsible antibiotic use and novel approaches to mitigate resistance development.

*Vibrio cholerae* is a unique gram-negative pathogen that occupies both aquatic environmental niches and human hosts during its life cycle. This dual lifestyle brings it into frequent contact with a wide range of microbial communities, including closely and distantly related species. Such interactions increase the likelihood of horizontal gene transfer (HGT), particularly involving MGEs like insertion sequences, integrons, and transposons. Among these, the IS*Aba125* element associated with the *bla*_NDM_ gene is of particular concern due to its strong linkage with carbapenem resistance—a growing threat in clinical settings globally. The presence of the *bla*_NDM_-*sh-ble* element in *V. cholerae* is particularly noteworthy given the bacterium’s genomic structure. Unlike many other gram-negative pathogens that typically possess a single circular chromosome, *V. cholerae* contains two chromosomes. This genomic organization not only reflects the evolutionary divergence of *V. cholerae* but also potentially offers multiple integration sites for MGEs. The possibility that IS*Aba125-bla*_NDM_-*sh-ble*-linked elements could be stably maintained across both chromosomes increases the risk of dissemination and persistence of antibiotic resistance within the species and across microbial populations. Furthermore, the high sequence similarity of the IS*Aba125-bla*_NDM_-*sh-ble* element in *V. cholerae* to similar elements found in other gram-negative pathogens, such as *Klebsiella pneumoniae*, *E. coli*, and *Acinetobacter baumannii*, suggests a shared evolutionary trajectory or common HGT mechanisms. This highlights the potential role of *V. cholerae* as both a reservoir and a vector for the spread of multidrug resistance genes across different ecological and clinical settings.

Our results demonstrated that IS*Aba125* enhances the spread of *bla*_NDM_ *sh-ble* under sublethal concentrations of antibiotics. Notably, the study reveals that IS*Aba125*-linked *bla*_NDM_ *sh-ble* genes can integrate into a diverse range of chromosomal loci, particularly near actively transcribed operons likely to take advantage of the elevated transcriptional activity for enhanced gene expression. Furthermore, this is the first study to systematically quantify the influence of the IS*Aba125* copy number on the stability and transmissibility of resistance genes both *in vitro* and *in vivo*.

## RESULTS

### Genome engineering of *V. cholerae* N16961 for monitoring IS*Aba125* mobility linked to *bla*_NDM_ *sh-ble*

**Fig 1:**
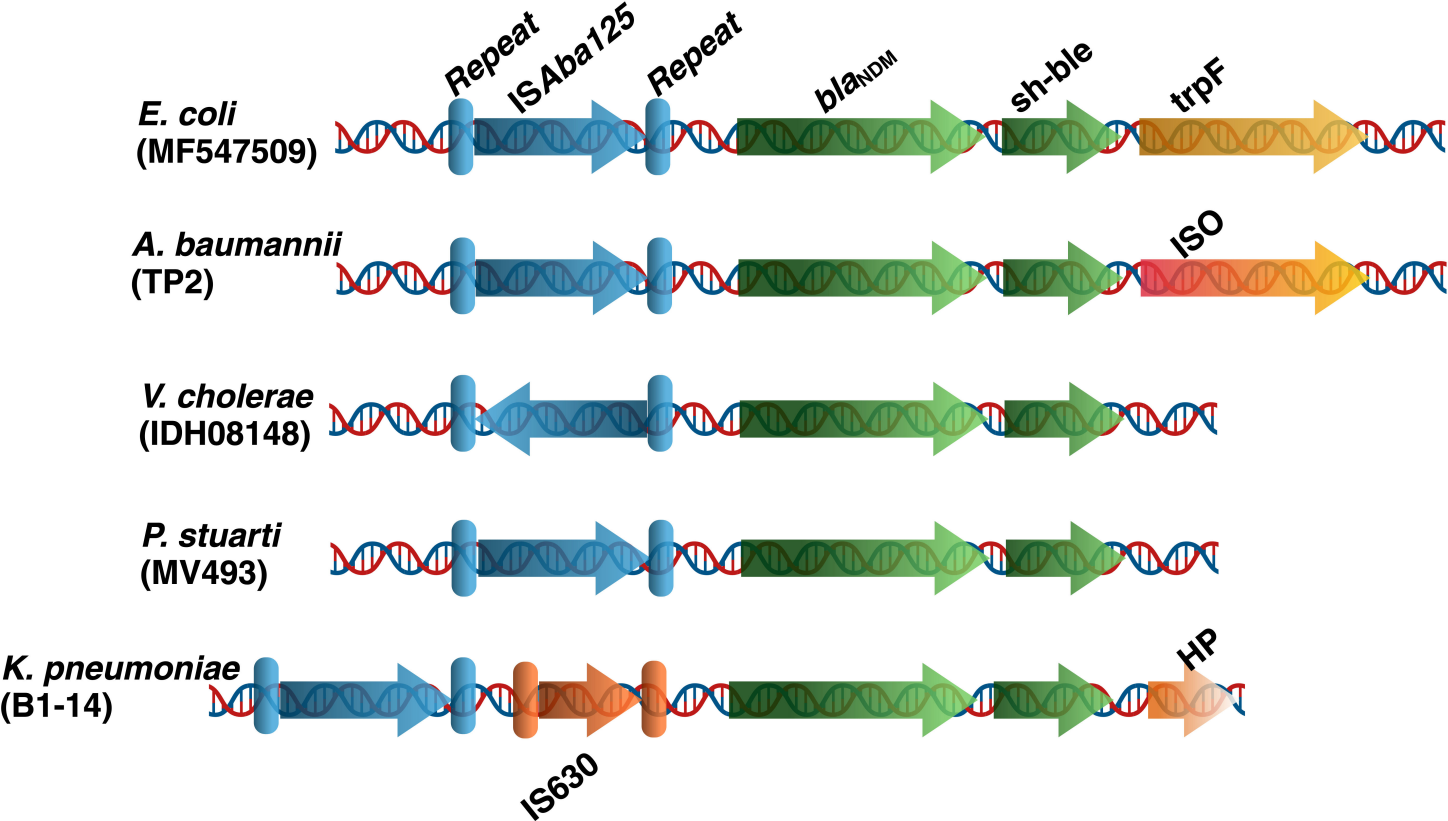
Schematic representation of the genetic organization of the IS*Aba125* element associated with the *bla*_NDM_ *sh-ble* gene cassette in high-priority bacterial pathogens. The IS*Aba125* element is genetically linked to the *bla*_NDM_ *sh-ble* gene cassette, illustrating the diverse genetic configurations across various pathogens.

In our previous work, through comprehensive sequencing and bioinformatic analysis, we identified a significant association between the insertion sequence IS*Aba125* and the *bla*_NDM_-*sh-ble* resistance gene cassette in several clinically important bacterial pathogens (2). As depicted in Fig. 1, we present a schematic representation of the diverse genetic organizations of the IS*Aba125* element in association with the *bla*_NDM_-*sh-ble* gene cassette across various bacterial species. Despite advancements in understanding the role of the transposase activity in the dissemination of IS*Aba125*-associated *bla*_NDM_ *sh-ble* resistance genes in *Enterobacteriaceae* and non-fermenter pathogens, the rate of mobility—encompassing both excision and integration of these elements under normal and antibiotic pressure—remains largely unexplored.

To address this, we initially constructed an array of genetically engineered *V. cholerae* reporter strains harboring the IS*Aba125* element linked with the metallo-beta-lactamase encoding *bla*_NDM_ gene both independently and in combination with other closely associated insertion sequences. For the construction of the reporter strains, we utilized a highly sensitive *V. cholerae* strain, N16961, devoid of resident IS*Aba125* or *bla*_NDM_ *sh-ble*. The recombinant vectors were constructed by amplifying IS*Aba125*, including its TIRs region, along with *bla*_NDM_ *sh-ble* resistance genes and its native promoter from the genome of different clinical isolates using specific set of primers (Table S1). These amplicons were then cloned into two vectors: a replicative temperature-sensitive suicide vector pFX524 having pSC101 origin of replication; and an in-house constructed integrative vector pBD60 (Table S2). The pFX524 vector was chosen for this study for two key reasons. First, it is temperature-sensitive, allowing for its removal at a non-permissive temperature and exhibiting low insertion bias, which facilitates random excision and integration of the insertion sequence along with the *bla*_NDM_ resistance gene into various genomic sites under temperature stress. Second, the pFX524 vector carries the R6K origin of replication, enabling plasmid replication solely in bacterial hosts capable of producing the lambda pir protein. Since the recipient cells do not produce the pir protein, the plasmid will not be maintained in the recipient strains, and only those recipients that have the insertion sequence-linked bla_NDM_ gene integrated into their chromosome grow under antibiotic selection. The advantage of using the pBD60 vector is that it integrates site specifically at the ‘dif’ site of the recipient bacterial chromosome and is stably expressed. The *dif* site is a conserved chromosomal locus where XerC and XerD recombinases mediate site-specific recombination to resolve chromosome dimers; it comprises two 11 bp binding regions flanking a 6 bp overlap site, facilitating strand exchange and integration of mobile genetic elements into bacterial chromosomes. The reporter strains conjugated with both recombinant pFX524 and pBD60 vectors were used to study the excision/integration frequency and *in vitro* stability of IS*Aba125-*linked *bla*_NDM_ *sh-ble* by plasmid curing and serial passaging experiments. The different reporter strains thus constructed are listed in Table S2.

### Transposition efficiency of IS*Aba125* in different genetic backgrounds

To examine the transposition efficiency of IS*Aba125* from plasmid to chromosome, a series of reporter strains (N:SK4, N:SK5, N:SK6, N:SK7, N:SK8, E:SK4, E:SK6, E:SK7 and E:SK8) carrying the *bla*_NDM_*sh-ble* resistance cassette linked to IS*Aba125* along with its right- (RIR) and left-inverted repeats (LIR) were constructed by cloning the desired DNA fragments into pFX524. Later on, the recombinant pFX524 vectors were subjected to a plasmid curing assay. Plasmid curing was done at a non-permissive temperature of 42°C. Although no transposition was observed from plasmid to chromosome in either *V. cholerae* or *E. coli* derivatives, differences in the plasmid curing efficiency between the two species were noted, with *V. cholerae* strains curing the plasmid faster than *E. coli* derivatives. This was confirmed, as the plasmid cured reporter strains did not grow in agar plates supplemented with either ampicillin, imipenem, or zeocin. Interestingly, a difference in the plasmid curing efficiency was identified between the *V. cholerae* and *E. coli* reporter strains, with the complete curing of plasmid confirmed at 96 h for the *V. cholerae* derivatives and 108 h for the *E. coli* derivatives.

### Effect of the copy number of transposases on *bla*_NDM_ *sh-ble* mobility

**Fig 2:**
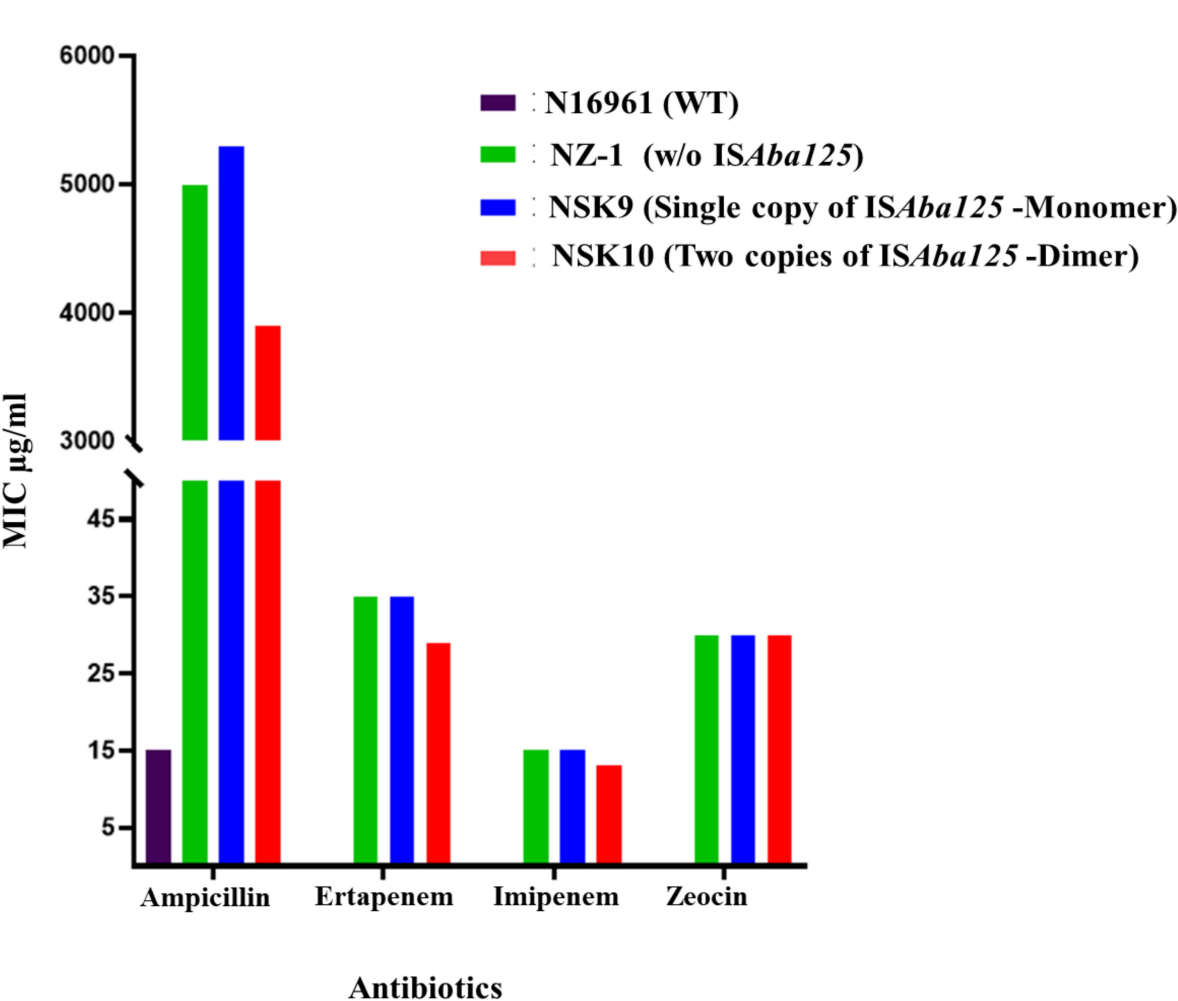
Minimum inhibitory concentration of beta-lactam and zeocin antibiotics against N16961 (WT), NZ-1, NSK9, and NSK10, which are N16961-derived reporter strains carrying either *bla*_NDM_ *sh-ble* or *bla*_NDM_ *sh-ble* along with single or two copies of IS*Aba125,* respectively.

A key finding from this study was the impact of the copy number of IS*Aba125* on the expression and excision frequency of the *bla*_NDM_ *sh-ble* gene. Reporter strains with a single copy of IS*Aba125* (monomer) exhibited higher resistance to antibiotics compared to strains with two copies (dimer) of IS*Aba125* (Fig. 2). Additionally, a gene excision assay was done using reporter strains at sub-lethal concentrations of different antibiotics to evaluate its role in transmissibility. The different antibiotics and their sub-lethal concentrations used in the experiment are listed in Table S3. Interestingly, it was observed that N:SK9, which harbored a single copy of IS*Aba125*, exhibited higher MIC than that of N:SK10, which had two copies of IS*Aba125* against the tested beta-lactam antibiotics (Fig. 2). Interestingly, while no significant difference was observed in the excision frequency between the two strains under normal conditions, the strain with a single copy of IS*Aba125* demonstrated a higher excision frequency when exposed to sublethal concentrations of antibiotics like mitomycin C, ciprofloxacin, and gentamicin (Table 1). This suggests that the copy number of IS*Aba125* affects its mobility and may influence the transmissibility of resistance genes. Spontaneous mutations which may lead to alteration in the catabolic pathway could potentially result in concomitant adaptation of phenotypic traits, such as gene inactivation, gene transfer, and changes in gene expression (11). Also, this could be due to auto-repression of the transposase at high concentration like those reported for Tn10, Tn5, and IS10 (12). Nevertheless, this report highlights the impact of the copy number of IS*Aba125* in the expression and excision frequency of the *bla*_NDM_ *sh-ble* gene.

**TABLE 1:** Excision frequencies of IS*Aba125* transposon-linked *bla*_NDM_ *sh-ble* in *V. cholerae* reporter strains NZ-1, N:SK9, and N:SK10 *in vitro^a^*

Antibiotic treatment	Strains (host)	Total CFU/mL	Screened colonies	Mean ± SD (%)	Excision frequency (%)
Untreated	NZ-1 (Tnp)	1.20 * 10^9^	216	ND^*c*^	ND
	NSK:9 (monomer)	2.98 * 10^9^	227	ND	ND
	NSK:10 (dimer)	2.03 * 10^9^	202	ND	ND
**Ciprofloxacin** **(CIP)^*b*^**	NZ-1 (Tnp)	2.49 * 10^9^	241	ND	ND
	**NSK:9 (monomer**)	1.05 * 10^9^	**319**	**1.5 ± 0.94**	**0.009114**
	NSK:10 (dimer)	1.20 * 10^9^	196	ND	ND
Doxycycline hyclate (DOX)	NZ-1 (Tnp)	1.33 * 10^9^	163	ND	ND
	NSK:9 (monomer)	1.06 * 10^9^	195	ND	ND
	NSK:10 (dimer)	1.02 * 10^9^	158	ND	ND
**Gentamicin** **(GEN)**	NZ-1 (Tnp)	1.22 * 10^9^	183	ND	ND
	**NSK:9 (monomer**)	1.09 * 10^9^	**144**	**1.0 ± 1.39**	**0.002642**
	NSK:10 (dimer)	1.05 * 10^9^	151	ND	ND
Kanamycin (KAN)	NZ-1 (Tnp)	1.11 * 10^9^	320	ND	ND
	NSK:9 (monomer)	2.01 * 10^9^	275	ND	ND
	NSK:10 (dimer)	2.02 * 10^9^	288	ND	ND
**Mitomycin C** **(MMC)**	**NZ-1 (Tnp**)	2.56 * 10^9^	**369**	**0.5 ± 0.81**	**0.004324**
	**NSK:9 (monomer**)	2.77 * 10^9^	**353**	**3.5 ± 1.98**	**0.008921**
	NSK:10 (dimer)	2.69 * 10^9^	246	ND	ND
Neomycin (NEO)	NZ-1 (Tnp)	2.19 * 10^8^	140	ND	ND
	NSK:9 (monomer)	2.32 * 10^8^	103	ND	ND
	NSK:10 (dimer)	2.00* 10^8^	110	ND	ND
Rifampin (RIF)	NZ-1 (Tnp)	2.36 * 10^9^	193	ND	ND
	NSK:9 (monomer)	2.81 * 10^9^	200	ND	ND
	NSK:10 (dimer)	3.22 * 10^9^	190	ND	ND
Tetracycline (TET)	NZ-1 (Tnp)	1.71 * 10^9^	145	ND	ND
	NSK:9 (monomer)	1.89 * 10^9^	113	ND	ND
	NSK:10 (dimer)	1.72 * 10^9^	110	ND	ND
Trimethoprim (TMP)	NZ-1 (Tnp)	1.06 * 10^9^	184	ND	ND
	NSK:9 (monomer)	1.91 * 10^9^	166	ND	ND
	NSK:10 (dimer)	1.22 * 10^9^	132	ND	ND

^*a*^ Tnp—transposon; monomer—single copy; dimer—two copies.

^*b*^ Boldface indicates experiments in which excision efficiency was determined.

^*c*^ “ND” indicates not detected.

### Identification and functional analysis of integration sites for IS*Aba125* in the *V. cholerae* genome

**Fig 3:**
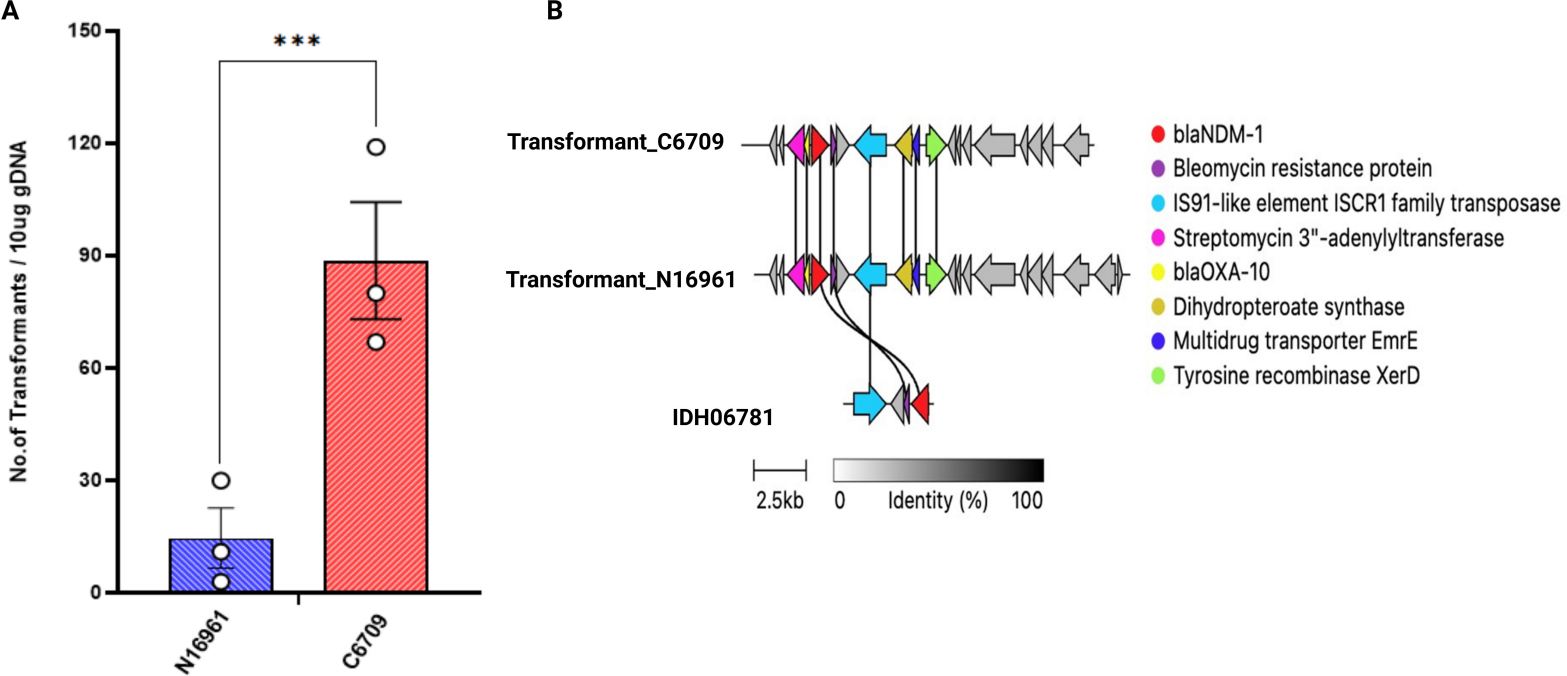
Transposition efficiency of the IS*Aba125-*linked *bla*_NDM_ *sh-ble* genes in different *V. cholerae* strains. (**A**) Chitin-induced natural transformation assay to investigate the transfer of the IS*Aba125*-linked *bla*_NDM_ *sh-ble* genes from *V. cholerae* IDH06781 strain to *bla*_NDM_-negative *V. cholerae* strains, HapR^+^ N16961 and C6709. (**B**) Genetic arrangement of the integration site of the IS*Aba125*-linked *bla*_NDM_ *sh-ble* in *V. cholerae* strains, HapR^+^ N16961 and C6709 transformants of the chitin assay. The IS*Aba*125 sequence was identified through BLAST in *bla*_OXA-10_ (yellow) sequence. Cluster alignments are drawn to scale using the Clinker tool version 0.0.26. Arrows indicate the direction of the open reading frame.

A chitin-induced natural transformation was conducted using *V. cholerae* clinical isolate C6709 and lab-generated N16961:HapR^+^ (streptomycin-sensitive) strains to investigate environmental acquisition of the IS*Aba125-bla*_NDM_-*sh-ble* element. Both HapR^+^ strains initially lacking the *bla*_NDM_-*sh-ble* element successfully acquired and chromosomally integrated the gene linked to IS*Aba125* following natural transformation with 10 µg of *V. cholerae* IDH06781 genomic DNA (Fig. 3A). Antibiotic susceptibility profiling confirmed that transformants exhibited resistance to β-lactam antibiotics, including penicillin, ampicillin, carbenicillin, and imipenem, compared to their wild-type counterparts (Fig. S1), supporting the role of natural transformation in disseminating clinically relevant resistance determinants in *V. cholerae* under environmental conditions.

To investigate the integration site of the IS*Aba125* element linked to the *bla*_NDM_*-sh-ble* gene cassette in the *V. cholerae* genome, N16961 HapR^+^ and C6709 transformants were subjected to whole-genome sequencing. The analysis of the genome sequences of both the transformants revealed the integration site of the *bla*_NDM_ *sh-ble* cassette into its chromosome. The analysis also identified a 130 bp nucleotide sequence homologous to the *attL* and *attR* attachment sites of IS*Aba125* in C6709’s genome, specifically between the 23S and 16S rRNA genes in chromosome I of the isolate (Fig. 3B). The integration site of the IS*Aba125-bla*_NDM_-*sh-ble* element in the N16961:HapR^+^ transformant could not be resolved using Illumina short-read sequencing due to the fragmented nature of the assembly and the small size of contigs carrying the resistance cassette. To precisely identify the chromosomal integration site, whole-genome sequencing was performed using Oxford Nanopore Technology (ONT), which generates long reads. These long reads were hybrid-assembled with the Illumina short reads to reconstruct a complete, high-quality genome assembly, which was then aligned to the *V. cholerae* N16961 reference genome. Unlike the C6709 transformant, where the IS*Aba125-bla*_NDM_-*sh-ble* element integrated into chromosome I, in the N16961 transformant, the cassette was found integrated into chromosome II proximal to the *infC-rpmI-rplT* operon. This operon encodes essential proteins involved in translation and ribosome assembly and is known to be actively transcribed in *V. cholerae*, similar to the rRNA operons—suggesting a potential preference for integration near transcriptionally active regions.

**Fig 4:**
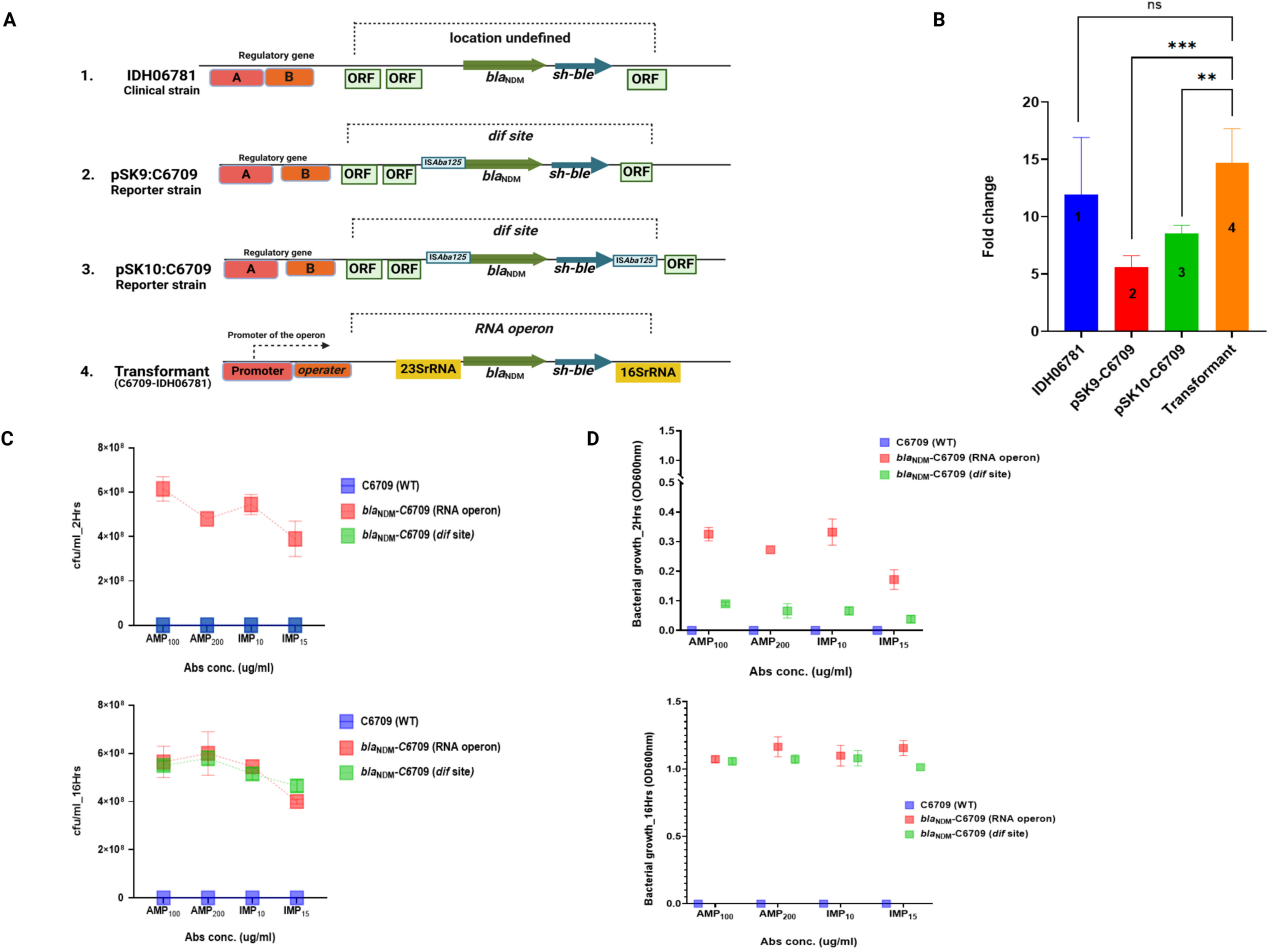
Activity of *bla*_NDM_ *sh-ble* in accordance with the site of integration into the genome of transformants. (**A**) Schematic representation of genomic locations of *bla*_NDM_ *sh-ble* in different *V. cholerae* strains (WT, reporter strains, and transformants). (**B**) Transcript analysis of the *bla*_NDM_ gene reporter strains and transformants having the *bla*_NDM_ *sh-ble* integrated at different genomic locations. The experiment was done in triplicates (*n* = 3). The expression levels of the target genes were normalized to that of the housekeeping gene, *recA*. Data were analyzed by unpaired *t*-test; values are expressed as mean ± SEM of all the three experiments using the GraphPad Prism version 10.9. (**C and D**) Graph illustrating the growth dynamics of C6709 (WT) and its transformants with *bla*_NDM_ *sh-ble* integrated at different genomic locations, as assessed by colony-forming units and turbidity assay (OD_600_) at 2 and 16 h in the presence of ampicillin and imipenem antibiotics.

To assess the functional consequences of this integration near actively transcribed operons, qRT-PCR analysis was conducted on C6709 transformants with the *bla*_NDM_ *sh-ble* linked to IS*Aba125* integrated either at the 23S rRNA-16S rRNA operon or at the ‘*dif’* site using specific primers *bla*_NDM_-F/*bla*_NDM_-R (Table S1). Results revealed a approximately six fold higher expression of the *bla*_NDM_ gene when integrated at the 23S rRNA–16S rRNA operon compared to the ‘*dif’* locus (Fig. 4A and B). This difference was further supported by CFU and turbidity assays under antibiotic pressure, where the transformant with integration at the 23S rRNA–16S rRNA operon exhibited significantly higher *bla*_NDM_ expression at earlier time points (Fig. 4C and D). These findings highlight the influence of the genomic integration site on the expression and functionality of resistance genes in *V. cholerae*.

### Analysis of sublethal concentration of antibiotics as an inducer of IS*Aba125* linked with *bla*_NDM_ *sh-ble* transmission

**Fig 5:**
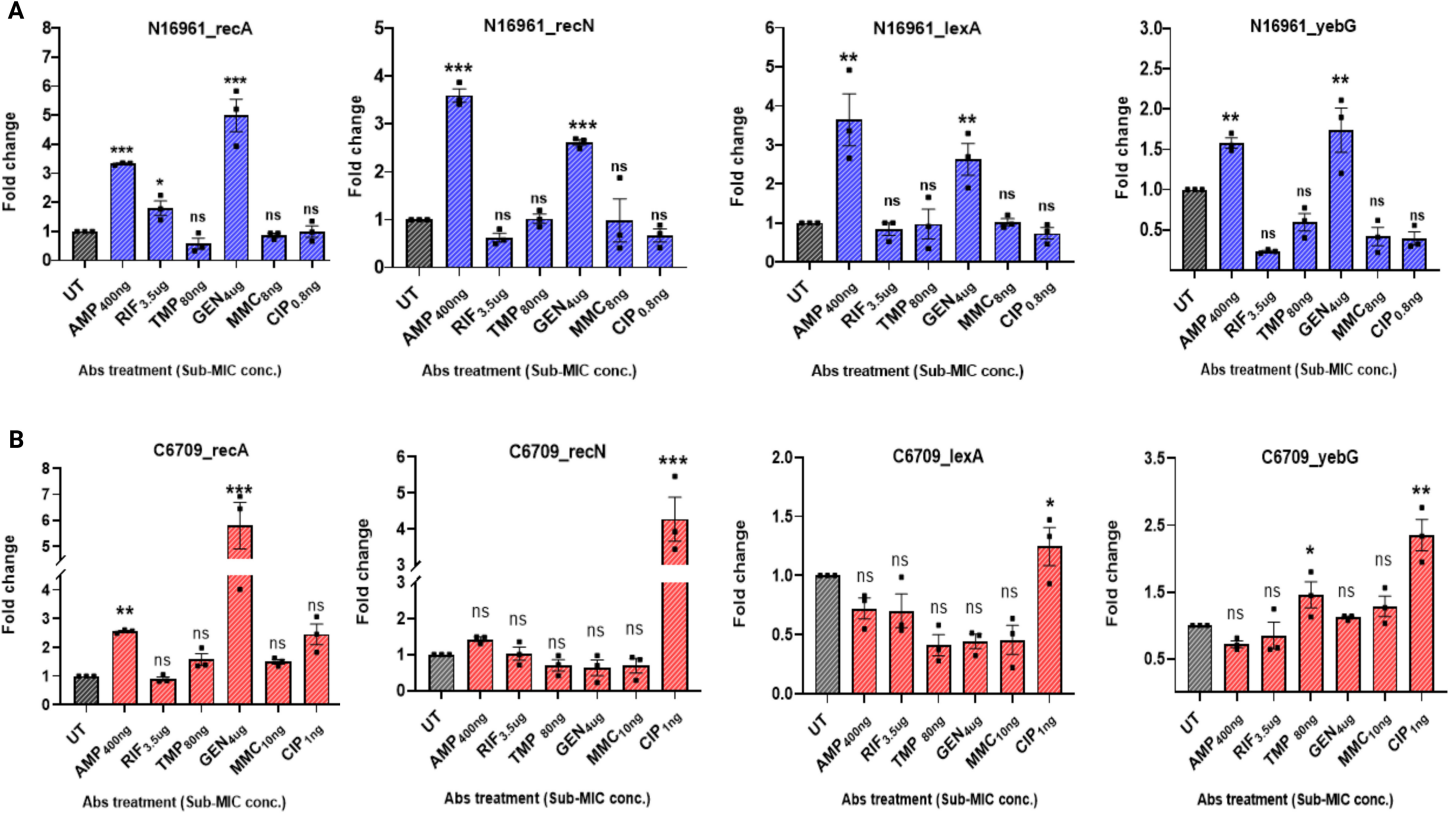
Relative gene expression levels of SOS genes in (**A**) N16961 and (**B**) C6709 under pressure of sublethal concentration of antibiotics compared to untreated (UT) control. The expression levels of the target genes (*recA*, *recN, lexA,* and *yebG*) were normalized to that of the housekeeping gene, *rpoB*. Statistical analysis was performed by unpaired *t*-test, and the data are presented as mean ± SEM (*n* = 3) (*P < 0.05, **P < 0.001, ***P < 0.0001) using GraphPad Prism version 10.9.

The role of sublethal antibiotic concentrations in inducing the transmission of IS*Aba125* linked with *bla*_NDM_ *sh-ble* was explored, focusing on their impact on the SOS response and gene transfer. Excision assays using the N:SK9 and N:SK10 reporter strains under sublethal antibiotic pressure (mitomycin C, ciprofloxacin, and gentamicin) demonstrated a significant increase in the excision frequency of IS*Aba125-*linked *bla*_NDM_. To further investigate, qRT-PCR analysis was performed to evaluate the expression of key SOS response genes (*recA, recN, lexA*, and *yebG*) in the presence and absence of sublethal antibiotic concentrations. The results revealed that exposure to antibiotics like ampicillin and gentamicin led to increased expressions of *recA*, *recN*, *lexA*, and *yebG* in N16961 (Fig. 5A and B). Additionally, exposure to ciprofloxacin and gentamicin enhanced the expressions of *recN*, *lexA, yebG*, and *recA*, respectively, in C6709.

To confirm that these antibiotic-induced changes were associated with the spread of IS*Aba125-*linked *bla*_NDM_ *sh-ble*, chitin-induced natural transformation assays were performed. The results indicated that sublethal concentrations of ampicillin and gentamicin enhanced the transformation efficiency and dissemination of *bla*_NDM_ *sh-ble* in N16961. This suggests that the SOS response activated by these antibiotics promotes the transmission of *bla*_NDM_ *sh-ble* (Fig. S2). However, no significant increase in transformation efficiency was observed for the C6709 strain under sublethal antibiotic pressure. These findings indicate that the effect of antibiotics in the spread of resistance genes primarily through SOS response activation may vary depending on the genetic makeup of the bacterial species.

### *In vivo* transmission and stability of IS*Aba125* linked with *bla*_NDM_ *sh-ble* in *V. cholerae* in a rabbit model

To assess the *in vivo* stability of the *bla*_NDM_ *sh-ble* resistance gene associated with IS*Aba125*, we used a rabbit ileal loop model. The use of the rabbit ileal loop model provides a physiologically relevant *in vivo* system for studying *V. cholerae* colonization, gene expression, and interaction with host tissues under conditions that closely mimic the human intestinal environment. In the context of studying the dynamics of IS*Aba125-bla*_NDM_-*sh-ble* element, this model is particularly valuable for assessing the stability, expression, and possible fitness costs or benefits of carrying the MGE in a host-associated context. This model also allows for the evaluation of the potential impact of the genetic element on the virulence or colonization ability of *V. cholerae*, which may differ significantly *in vivo* compared to *in vitro* observations. Given the global health implications of *bla*_NDM_ dissemination, understanding how such elements behave and persist within *V. cholerae* during infection can provide critical insights into the transmission dynamics and evolutionary fitness of antibiotic-resistant strains.

Reporter strains carrying the *bla*_NDM_*-sh-ble*-IS*Aba125* construct were inoculated into ligated rabbit ileal loops and incubated for 16–18 h. The fluid from the ileal loops was collected, and CFUs were counted by plating on LA agar and TCBS selective plates. The presence of *bla*_NDM_ *sh-ble* was confirmed through resistance profiling and PCR amplification using gene-specific primers.

*In vivo* experiments revealed a higher frequency of *bla*_NDM_ *sh-ble* loss in the NZ-1 strain, which lacks the transposon, compared to strains containing the transposon (Table 2). Notably, the NSK:10 strain, which carries two copies of IS*Aba125*, showed no gene loss, consistent with *in vitro* findings. Gene loss frequencies were determined by plating on selective media containing ampicillin, zeocin, and streptomycin. The frequency of *bla*_NDM_ *sh-ble* loss was significantly higher in the NZ-1 strain under *in vivo* conditions, as opposed to *in vitro* conditions. Conversely, the NSK:9 strain exhibited greater gene loss *in vitro* than *in vivo*. These results suggest that the presence of multiple IS*Aba125* copies enhances *bla*_NDM_ *sh-ble* stability *in vivo*.

**TABLE 2:** Excision frequencies of the IS*Aba125* transposon-linked *bla*_NDM_ *sh-ble* in *V. cholerae* reporter strains NZ-1, N:SK9, and N:SK10 *in vivo^a^^,^^b^*

*In vivo*	Strains (host)	Total *V. cholerae* CFU/mL	Total *V. cholerae* screened colonies	Mean ± SD (%)	Excision frequency (%)
Infection incubation period 18 h	NZ-1 (Tnp)	2.8 * 10^7^	1230	14 ± 2.276	12.300000
	NSK:9 (monomer)	1.1 * 10^7^	951	1 ± 0.210	1.729091
	NSK:10 (dimer)	7.0 * 10^7^	1110	ND	ND

^*a*^ Tnp—transposon; monomer—single copy; dimer—two copies.

^*b*^ “ND” indicates not detected.

## DISCUSSION

Mobile genetic elements (MGEs) are pivotal in the horizontal transfer of resistance genes among bacterial populations. Among these, insertion sequences (ISs) are the simplest and most commonly encountered MGEs, playing a significant role in the dissemination of resistance genes. Notably, previous studies have highlighted a strong genetic association between the insertion sequence IS*Aba125* and the carbapenemase gene *bla*_NDM_. IS*Aba125* has been identified as a key contributor to the rapid spread of *bla*_NDM_, underscoring its critical role in the expansion of antimicrobial resistance (2, 4, 13). This study aimed to investigate the mobility and integration of the IS*Aba125* element linked to the *bla*_NDM_ sh-ble resistance gene cassette, specifically in *V. cholerae* N16961. Our previous work demonstrated a strong genetic association between IS*Aba125* and *bla*_NDM_ *sh-ble* in clinically significant pathogens (2, 14). In this study, we sought to explore the dynamics of IS*Aba125* mobility, focusing on transposition efficiency, integration sites, and the influence of antibiotic pressure on gene transfer.

*V. cholerae* was strategically selected as the model organism for investigating the transmission dynamics of the IS*Aba125*-associated *bla*_NDM_*-sh-ble* element due to multiple compelling factors. Initially, the direct detection of this genetic linkage in *V. cholerae* isolates from diarrheal patients provided a clinically relevant foundation for the study. Furthermore, emerging evidence indicates that *V. cholerae* possesses an enhanced ability to acquire and integrate novel antimicrobial resistance determinants, underscoring its role as a dynamic genetic reservoir. Its ecological niche in aquatic environments characterized by extensive microbial diversity and frequent HGT events further positions *V. cholerae* as a critical conduit for resistance gene dissemination. The clinical significance of *V. cholerae* as the causative agent of cholera amplifies the potential public health impact of resistance development within this species. Importantly, the well-characterized and experimentally tractable HGT systems in *V. cholerae* provide a robust and versatile model to dissect the molecular mechanisms underlying the acquisition, integration, and spread of IS*Aba125*-linked *bla*_NDM_*-sh-ble*. Collectively, these features establish *V. cholerae* as an ideal chassis for elucidating the transmission dynamics of this mobile genetic element, with direct implications for understanding and mitigating antimicrobial resistance in both environmental and clinical contexts.

We successfully engineered the genome of the *V. cholerae* strain, N16961, to develop reporter strains that facilitate the monitoring of the IS*Aba125* mobility linked with the *bla*_NDM_*-sh-ble* resistance gene. This innovative approach not only provides a valuable tool for investigating the dynamics of antibiotic resistance elements but also enhances our understanding of the mechanisms underlying the spread of *bla*_NDM_ among enteric bacteria.

Our analysis of the transposition efficiency of IS*Aba125* across different genetic backgrounds revealed no significant variability, as none of the *V. cholerae* N16961 and *E. coli* reporter strains carrying different combinations of insertion sequences, including IS*3*/IS*Aba14,* IS*911,* IS*91,* and IS*630*, along with IS*Aba125*, exhibited transposition of the resistance cassette from plasmid to the chromosome. However, a difference was observed in the plasmid stability among the different *V. cholerae* and *E. coli* reporter strains. Plasmids were less stable in the *V. cholerae* strain possibly due to the poor domestication of the exogenous plasmid in the isolate due to the presence of two chromosomes (15). Understanding these interactions is vital for predicting the potential for HGT, particularly in clinically relevant strains.

However, our investigation into the effect of the transposase copy number on the mobility and activity of IS*Aba125* linked with *bla*_NDM_ *sh-ble* demonstrated that different transposase copy numbers modulate the transposition frequency and activity of the associated resistance genes. This modulation of transposase activity might serve as a strategic approach to control the dissemination of *bla*_NDM_ in pathogenic bacteria. The identification and analysis of integration sites for IS*Aba125* within the *V. cholerae* genome revealed that it can integrate in a diverse array of loci, emphasizing the plasticity of bacterial genomes in accommodating MGEs. Understanding these integration hotspots is crucial for mapping the pathways of antibiotic resistance gene dissemination and for developing predictive models of resistance evolution. Such insights may inform the design of effective surveillance strategies in healthcare settings.

*In vivo* stability of the *bla*_NDM_ *sh-ble* cassette linked with IS*Aba125* was assessed using a rabbit ileal loop model, revealing that strains with multiple copies of IS*Aba125* exhibited the highest stability of the *bla*_NDM_ *sh-ble* gene compared to strains with a single copy or no transposase. This finding suggests that the presence of multiple copies of IS*Aba125* enhances gene stability, even in the absence of antibiotic pressure. It is likely that the transposase efficiently reintegrates the excised cassette before cell division, maintaining stability. In contrast, strains lacking transposase exhibited no integration events, leading to a higher frequency of *bla*_NDM_ *sh-ble* cassette loss. However, the precise molecular mechanisms behind the increased excision and loss frequencies in the absence of transposase remain unclear. Similarly, the exact mechanism by which the transposase copy number influences the transposition efficiency could not be fully elucidated in the present study. These observations highlight the need for further investigations to better understand the dynamics of IS*Aba125* mobility and integration, which could offer valuable insights into controlling the spread of antibiotic resistance gene cassettes in clinically relevant bacterial species. Future studies could focus on evaluating the potential auto-repression of IS*Aba125*. Understanding these mechanisms may be crucial for developing strategies to mitigate the transmission of antimicrobial resistance.

### Conclusion

In conclusion, this study reveals the complex dynamics of *bla*_NDM_ mobility linked with IS*Aba125*, highlighting the role of antibiotic exposure in modulating transposition efficiency and genetic adaptation. Our findings demonstrate that IS*Aba125*’s behavior is influenced by genetic context, host background, and antibiotic pressure, which can enhance the mobility of resistance genes. The integration of IS*Aba125* in the *V. cholerae* genome further suggests its potential for horizontal gene transfer, functioning similarly to site-specific recombination systems. These insights underscore the importance of targeting IS*Aba125* to combat the spread of antibiotic resistance and emphasize the need for ongoing research to develop effective strategies against resistant pathogens.

## MATERIALS AND METHODS

### Bacterial strains, plasmids, and growth conditions

The bacterial isolates used in this study are listed in Table S4. For the liquid culture, the strains were grown in Luria-Bertani broth (LB) or Mueller Hinton broth (MHB) at 37°C in a shaker with 180 RPM, while LB agar plates were used for the solid culture. Bacterial strains were preserved at −80°C in LB containing 15% sterile glycerol. Antibiotics (Sigma-Aldrich, USA) were used at the following concentrations, unless mentioned otherwise: ampicillin: 100 µg mL^−1^; kanamycin: 40 µg mL^−1^; streptomycin: 100 µg mL^−1^; and chloramphenicol: 3 µg mL^−1^ for *V. cholerae*. Before the initiation of any experiment, bacterial strains were freshly inoculated from the −80°C stock. Bacterial growth was monitored by measuring the optical density (OD) at 600 nm using a biospectrophotometer (Eppendorf, Hamburg, Germany).

### Molecular biological methods

Standard molecular biological methods for chromosomal and plasmid DNA preparations, restriction enzyme digestion, DNA ligation, bacterial transformation, conjugation, agarose gel electrophoresis, etc., were followed, unless stated otherwise. Restriction and nucleic acid-modifying enzymes were purchased from New England Biolabs, Inc. (NEB, USA) and used as directed by the manufacturer. The T4 DNA ligase enzyme used in this study was procured from NEB (USA). Ligation reactions were carried out essentially as directed by the manufacturer.

### Construction of plasmids and reporter strains

The recombinant plasmids were constructed by the PCR amplification of the *bla*_NDM_ *sh-ble* IS*Aba125* ORF with its natural promoter (size 2.7 kb) alone and in conjunction with other insertion sequences, as listed in Table S2. The genomic DNA of clinical isolates listed in Table S4 harboring the IS*Aba125* linked *bla*_NDM_ was used as the template. The amplicons were purified, restriction enzyme digested, and ligated into vectors pFX524 and pBD60 (16). Furthermore, the ligation mixture was transformed into FCV14 *E. coli* cells, and the positive transformants with the recombinant plasmid were selected on ampicillin (100 µg mL^−1^) plates. The recombinant plasmid was then isolated from the positive transformants and further transformed into the intermediate host *E. coli* β2163 to conjugate it with N16961 and *E. coli* ATCC 25,922 cells to generate the genetically engineered reporter strains. The conjugation plate was supplemented with 0.3 mM DAP for selecting β2163 transformants. Exponentially growing (optical density at 600 nm, ∼0.3) donor and recipient cells were mixed in a 1/2 ratio and incubated at 30 and 37°C overnight for the replicative (pFX524) and integrative vectors (pBD60), respectively. The transconjugants were selected on agar plates supplemented with ampicillin and zeocin at concentrations of 100 and 25 µg mL^−1^.

### Transposition assays

To understand the transposition efficacy of IS*Aba125-*linked *bla*_NDM_*sh-ble* from plasmids, the transposition assay was performed as previously described with some modifications (17). Briefly, N16961 and *E. coli* ATCC 25922 reporter strains N:SK4, N:SK5, N:SK6, N:SK7, N:SK8, E:SK4, E:SK6, E:SK7, and E:SK8 with the recombinant pFX524 plasmid were initially grown overnight in the LB containing 100 µg mL^−1^ ampicillin. Overnight cultures were then diluted by a factor of 100 in the absence of antibiotic selection and grown at 42°C, with shaking for approximately 300 generations. At every 18 h, the cultures were serially diluted and spread on LB agar plates and incubated overnight at 30°C. The next day, around 100 colonies were picked at random, patched onto selective media containing ampicillin, and grown overnight at 30°C. After incubation, colonies with optimum growth were counted and confirmed by *bla*_NDM_-specific PCR, and the transposition frequency was evaluated. The transposition of IS*Aba125-*linked *bla*_NDM_ from plasmid togenome was confirmed by two sets of PCR reactions. A PCR using plasmid-specific primers was performed to confirm the successful elimination of the plasmid at non-permissive temperature from the bacterial cell. In addition, a PCR using *bla*_NDM_ specific primers was performed to confirm a successful integration of the genome into the bacterial chromosome. Additionally, the growth of the plasmid-cured reporter strains was assessed on agar plates supplemented with either ampicillin (100 µg mL^−1^), imipenem (10 µg mL^−1^), or zeocin (25 µg mL^−1^).

### Antibiotic susceptibility testing and minimum inhibitory concentration (MIC) assay

The antibiotic susceptibility (AST) profiling of the wild type and transformants was performed by disc diffusion and broth microdilution assays. The MIC of reporter strains and transformants of the chitin assay was determined by broth dilution method of antimicrobial susceptibility testing according to CLSI guidelines (18). Briefly, overnight bacterial culture grown in the MHB medium was diluted at 1:100 in fresh MHB and cultured at 37°C with shaking at 180 RPM to an optical density of OD_600_ 0.5. Furthermore, the cells were diluted at 1:1,000 in fresh MHB again, and 100 µL of this diluted culture was dispensed into each well of a 96-well microtiter polystyrene plate. A series of twofold dilutions of an antibiotic was added into these wells, and the mixtures were incubated at 37°C for 16–18 h.

### Chitin-induced natural transformation

Chitin-induced natural transformation was carried out as previously described with few modifications (19). Briefly, the recipient HapR^+^ *V. cholerae* bacterial strains N16961 (HapR^+^, str^s^) and C6709 were grown on 50–80 mg autoclaved chitin flakes (Sigma cat. C9213) immersed in M9 medium supplemented with MgSO_4_ and CaCl_2_ for 16–20 h at 30°C. Next day, the spent media were replenished with fresh M9 media with MgSO_4_ and CaCl_2_, and the donor DNA, 10 µg of genomic DNA from a highly resistant clinical *V. cholerae* isolate, IDH06781, was added as the transforming material. The genomic DNA of the *V. cholerae* reporter strain, NZ-1, with *bla*_NDM_ *sh-ble* cassette was used as the positive control. Cells were further incubated for 24 h and subsequently detached from the chitin by vigorous vortexing for 30 s to 1 min. The transformants were selected on LB agar plates supplemented with ampicillin (100 µg mL^−1^) antibiotic. The antibiotics and their sublethal concentrations against the *V. cholerae* HapR + str^s^ N16961 and str^r^ C6709 strains used in chitin assay are presented in Table S5.

### Quantitative RT-PCR (qRT-PCR) analysis of SOS (save our soul) gene expression

Real-time reverse transcriptase PCR was performed to detect the differential expression of the *bla*_NDM_ gene and the SOS response genes (*recA*, *recN*, *lexA*, and *yebG*) in the transformants of chitin-induced natural transformation. The primer pairs were designed using Primer 3 software version 0.4.0. For sample preparation, three fresh colonies of each strain were inoculated separately into LB broth and incubated overnight at 30°C with shaking condition (180 rpm). The next day, overnight cultures were diluted 100-fold in 5 mL of fresh LB broth and further grown to mid-log for 1–2 h (OD_600_ = 0.3) at 30°C with shaking at 180 RPM. At this point, sublethal concentrations of antibiotics were added, and the cells were grown to late-log phase to reach OD_600_ = 0.7–0.8 (∼2 × 10^8^ CFU/mL). One milliliter from each culture was aliquoted, and the cultures were washed with cold PBS. Furthermore, the bacterial cells were harvested by centrifugation at 5,000 RPM before RNA extraction using Trizol. First-strand cDNA was synthesized using Qiagen’s QuantiTect Reverse Transcription Kit from a 100 ng RNA template. Real-time PCR was carried out in a two-step method on a QuantiStudio 6 Real-Time PCR Machine of Thermo Fisher Scientific using the Power Track SYBER Green Master Mix (Applied Biosystem). The primers used for qRT-PCR are listed in Table S1. The expression of genes was normalized to housekeeping gene *rpoB* as internal control. Each assay was performed in technical triplicates and biological duplicates.

### Persistence assay

To test the stability of IS*Aba125-*linked *bla*_NDM_ in the genome of *V. cholerae* isolates, reporter strains N:SK9, N:SK10, and NZ-1 were used, which had the gene cassette integrated into the ‘*dif*’ loci using the pBD60 vector. Serial passaging of the reporter strains in the presence and absence of a sublethal concentration of antibiotics was done to evaluate the excision frequency of the resistance gene linked to IS*Aba125*. Briefly, overnight cultures of the reporter strains were diluted 100-fold in 5 mL of fresh LB medium and incubated in the presence and absence of sublethal concentration of antibiotics at 37°C with shaking at 180 RPM/min for 16 h. The next day, 100 µL of the cultures from each tube was further diluted in fresh LB broth with the same conditions and incubated further. The serial passaging was continued every 12 h and, at each passage, 100 µL of the cultures was also serially diluted and plated on LB agar medium having appropriate antibiotics to determine the CFU. To estimate the excision frequency *in vitro*, at least 100 colonies derived from each culture were plated onto fresh nutrient agar with or without ampicillin plus zeocin. Clones that had become ampicillin 100 µg mL^−1^ plus zeocin 12.5 µg mL^−1^-sensitive were screened by PCR using primers listed in Table S1. Nine different antibiotics primarily acting on cell wall synthesis and permeability were selected for the survival assay. The sublethal concentrations of the antibiotics were optimized using the double-dilution method.

### Wholegenome sequencing and bioinformatics analysis

The WGS of the N16961 (HapR^+^, str^s^) and C6709 transformants selected from the chitin-induced natural transformation of the IDH06781 genome was performed in-house by a high-throughput Illumina MiSeq sequencing platform using the Nextera XT DNA Library Preparation Kit (Illumina, Inc.). To obtain the long-read sequences of the N16961:HapR^+^ transformant, whole-genome sequencing was performed using Oxford Nanopore Technology. The genomic DNA was end-polished and A-tailed (NEBNext Ultra II End Repair Kit, New England Biolabs, MA, USA). The end-prepared samples were barcoded using Blunt TA ligase master mix (M0367L). The equimolar concentration of the barcoded sample was pooled, and the library was prepared by ligating sequencing adapters (SQK-NBD114.96 Kit (Oxford Nanopore Technology, UK) onto double-stranded DNA fragments using NEB Quick T4 DNA ligase (New England Biolabs, MA, USA). After purification with AMPure XP beads, the prepared library was ready for library quality control. 

### The experimental procedures of the DNA library preparation

The library was checked with Qubit for quantification and pooled and sequenced on Nanopore PromethION System (PromethION P24 and Data Acquisition Unit, ONT, Oxford, UK) using a PromethION flow cell (FLO-PRO114M) according to effective library concentration and the data amount required. High-quality raw reads were used for assembly using a hybrid assembly pipeline Unicycler version v0.4.8 (20). The assembled genome quality was assessed for contamination using the CheckM Program version v1.1.3 (21). Genomes with contamination levels < 5% were included for analysis. A hybrid genome assembly was performed using Unicycler, which combines both short- and long-read sequencing data to generate high-quality contigs. The resulting *de novo* assembled genome was aligned against the reference *V. cholerae* strain N16961 using minimap2 (v2.26). The SAM file obtained from the alignment was subsequently converted to a sorted BAM format and indexed using SAMtools. To validate the presence and determine the precise coordinates of the *bla*_NDM_ insertion, we conducted a nucleotide BLAST (BLASTn) of the gene against the assembled genome. Then, Prokka and Rapid Annotation using Subsystem Technology (RAST) version 2.0 were used for the functional annotation of the draft assembly, enabling the identification of coding sequences and the annotation of the flanking regions surrounding the insertion (22).

### Rabbit ileal loop assay

The rabbit ileal loop assay was carried out on young New Zealand white rabbits weighing approximately 2.0 kg. The rabbits were starved for 24 h before the surgery was done, but water was given *ad libitum*. *V. cholerae* strains N16961, NZ-1, NSK:9, NSK:10, and NSK12 were cultured in LB until the mid-log phase (OD_600_ 0.4 to 0.5) corresponding to 10^8^ CFU/mL, followed by washing with sterile phosphate-buffered saline (PBS) and resuspended in 1 mL PBS. After administering anesthesia (ketamine and xylazine, as per bodyweight) and performing a laparotomy, the ileum of the rabbits was sectioned into separate ligated loops, each measuring approximately 10 cm and interloops of 2 cm. *V. cholerae* strains N16961, NZ-1, NSK:9, NSK:10, or NSK12 were then inoculated into the ligated loops separately, and the loop containing only PBS was used as the negative control. The intestine was repositioned into the peritoneum, and the abdomen was sutured back. After 16 to 18 h post-infection, the rabbits were euthanized; the abdomen was opened; and the length and volume of fluid that had accumulated in each loop were measured. The ileal fluid accumulation (FA) ratio was calculated by the following formula: volume of fluid accumulated (mL)/length of intestine (centimeters). The FA ratio of greater than 1 was considered a strong positive response, while that less than 0.2 indicated a negative response. To determine the *in vivo* excision frequency, the ileal fluid was first enriched overnight in LB broth at 37°C with shaking at 180 RPM. The following day, serial dilutions (10^0^–10^6^) were performed and plated onto LA plates. Subsequently, 1,500 colonies from each culture were screened onto fresh agar plates with or without ampicillin and zeocin. Clones that had become sensitive to ampicillin 100 µg mL^−1^ and zeocin 12.5 µg mL^−1^ were further confirmed by PCR using primers listed in Table S1. The experiment was done in biological triplicate in three rabbits, and the average of the independent results was compiled.

### Statistical analysis

All statistical analyses were performed using GraphPad Prism version 10.9. (GraphPad Software, Inc.) and display the experimental data of at least two independent experiments performed in triplicate, displaying the mean and the standard error of mean (SEM) throughout the manuscript, except for animal-related experiments, which display mean ± 95% confidence intervals. Student’s *t*-test was performed for analyzing the results. Statistical significance was accepted at *P* < 0.05. ^∗^*P* < 0.05, ^∗∗^*P* < 0.01, and ^∗∗∗^*P* < 0.001.

## Data Availability

Whole genome sequences are available in the National Centre for Biotechnology Information (https://www.ncbi.nlm.nih.gov/) under Bioproject ID PRJEB90998 and Indian Biological Data Centre (https://ibdc.dbtindia.gov.in) under accession number INRP000349.

## SUPPLEMENTAL MATERIAL

Supplemental tables and figure legends jb.00133-25-s0001.pdf Tables S1 to S5 and legends for Fig. S1 and S2.

Figure S1 jb.00133-25-s0002.tiff Heat map showing antibiotic susceptibility profiles of *V. cholerae*.

Figure S2 jb.00133-25-s0003.tiff Transformation efficiency of N16961 and C6709 in different antibiotic pressures.

ASM does not own the copyrights to Supplemental Material that may be linked to, or accessed through, an article. The authors have granted ASM a non-exclusive, world-wide license to publish the Supplemental Material files. Please contact the corresponding author directly for reuse.
