# Supplementary material for: Investigating the molecular transmission dynamics of blaNDM in antibiotic-selective environments
Source: J Bacteriol. 2025 Aug 11;207(9):e00133-25. doi: 10.1128/jb.00133-25 (PMC12445084; doi:10.1128/jb.00133-25)
Supplement: Supplemental tables and figure legends — Tables S1 to S5 and legends for Fig. S1 and S2. [file jb.00133-25-s0001.pdf]

Table S1: Oligonucleotides used in this study.

Oligo-nucleotide	Use	Sequence (5'-3')
1393F	Cloning of *bla*$_{NDM}$, *sh-ble*+ IS*Aba125* region	CGTCTAGAAGAACACCAACCCGGCATAG
1394R	Cloning of *bla*$_{NDM}$, *sh-ble*+ IS*Aba125* region	CGAAGCTTTCTTGGCCGTGCTGGGTTAG
1431F	Cloning of Tn + *bla*$_{NDM}$, *sh-ble* repeat region of IS91	GCAAGCTTCGATGCGGCGCCAGTGGACG
1426 R	Cloning of Tn + *bla*$_{NDM}$, *sh-ble* repeat region of IS91	GCTCTAGACACGTTCGTATCGGTGCGTTC
1525F	Cloning of *bla*$_{NDM}$, *sh-ble*+IS630 + IS*Aba125* region	CGAAGCTTGATGCCTCGGAGGAAACTG
1526-R	Cloning of *bla*$_{NDM}$, *sh-ble*+IS630 + IS*Aba125* region	GCTCTAGAAACACCAACCCGGCATAGTC
1486-F	Cloning of *bla*$_{NDM}$, *sh-ble*+ IS*Aba125* +IS*Aba14*+IS911	GCGGTACCTTCGGGCGATTCCGAACCGTGC
1432R	Cloning of *bla*$_{NDM}$, *sh-ble*+ IS*Aba125* +IS*Aba14*+IS911	GCACTAGTCTTGTAGCGGGCTTAATTGG
1395F	Cloning of IS*Aba125* region	GCTCTAGACAGACATTCGGTGCGAGCTG
1396R	Cloning of IS*Aba125* region	CGGGTACCGATGCCTCGGAGGAAACTGC
1727-F	Cloning of *bla*$_{NDM}$, *sh-ble*+ IS*Aba125* region	CGCCGCGGAGAACACCAACCCGGCATAG
1728-R	Cloning of *bla*$_{NDM}$, *sh-ble*+ IS*Aba125* region	CGTCTAGACATGGCGATATCACGGATAG
1731-F	Cloning of IS*Aba125* region	GCCCGCGGACCGGGTGCATAATATTG
1732-R	Cloning of IS*Aba125* region	GCCCGCGGGTTGAACGTTGCCTAAGAG
968 F	*rpoB* - Housekeeping gene	CTGTCTCAAGCCGGTTACAA
969 R	*rpoB* – Housekeeping gene	ATGCGATCTGTCGTGCTATC
905 F	*recA* - SOS gene, qPCR	GCAATCAAAGAAGGCGAAGAAG
906 R	*recA* - SOS gene, qPCR	GGCCATACATGATCTGAGTGTT
1651-F	*lexA* - SOS gene, qPCR	AGTTTCGGCTTCTTCTTCGT
1652-R	*lexA* - SOS gene, qPCR	CCGAAATTGCTAAAGAGCTG
1653-F	*recN* - SOS gene, qPCR	ATACCCACGTCAACTTCATC
1654-R	*recN* - SOS gene, qPCR	TGCCGTGACTTTCTTAGTCT
1655-F	*yebG* - SOS gene, qPCR	CTGGGTAAAAGTGCTCTGAT
1656-R	*yebG* - SOS gene, qPCR	CTCATCGTCATCACTGTCTG
1804F	Expression of *bla*$_{NDM}$ gene, qPCR	TTGGCATAAGTCGCAATCCC
1805R	Expression of *bla*$_{NDM}$ gene, qPCR	GTTTGATCGTCAGGGATGGC
684R	Amplification of *bla*$_{NDM}$ *sh-ble*	GCAGTACTTCAGTCGGGGTTCTGGATCAG
1295F	Amplification of *bla*$_{NDM}$ *sh-ble*	GGCCTCGCATTTGCGGGG

Table S2: Relevant plasmids used in the study, transposons amplified and reporter strains constructed with their genotypes.

Plasmids, Transposons & Strains	Genotype & Phenotype	Source/ reference
**Plasmids**		
pFX524	pSC101 *ori*, Amp^r	(23)
pBD60	Zeo^r, Cmp^r	Lab stock
pSB49	Zeo^r, Cmp^r	Lab stock
pSK4	IS*Aba125*, *bla*$_{NDM}$, *sh-ble,* Amp^r, Zeo^r, Imp^r	This study
pSK5	Repeat regions, IS*Aba125**, *bla*$_{NDM}$, *sh-ble,* Amp^r, Zeo^r, Imp^r	This study
pSK6	IS*Aba125*, IS630, *bla*$_{NDM}$, *sh-ble,* Amp^r, Zeo^r, Imp^r	This study
pSK7	IS*Aba125*, IS3/IS911, IS*Aba14*, *bla*$_{NDM}$, *sh-ble,* Amp^r, Zeo^r, Imp^r	This study
pSK8	IS*Aba125*, *bla*$_{NDM}$, *sh-ble,* IS*Aba125*, Amp^r, Zeo^r, Imp^r	This study
pSK9	IS*Aba125*, *bla*$_{NDM}$, *sh-ble,* Amp^r, Zeo^r, Imp^r	This study
pSK10	IS*Aba125*, *bla*$_{NDM}$, *sh-ble,* IS*Aba125*, Amp^r, Zeo^r, Imp^r	This study
pSK12	pBD60*,* Zeo^r, Cmp^r	This study
**Reporter strains**		
N:SK4	pSK4: *V. cholerae* N16961	This study
N:SK5	pSK5: *V. cholerae* N16961	This study
N:SK6	pSK6: *V. cholerae* N16961	This study
N:SK7	pSK7: *V. cholerae* N16961	This study
N:SK8	pSK8: *V. cholerae* N16961	This study
N:SK9	pSK9: *V. cholerae* N16961	This study
N:SK10	pSK10: *V. cholerae* N16961	This study
N:SK12	pSK12: *V. cholerae* N16961	This study

E:SK4	pSK4: *E. coli* ATCC 25922	This study
E:SK6	pSK6: *E. coli* ATCC 25922	This study
E:SK7	pSK7: *E. coli* ATCC 25922	This study
E:SK8	pSK8: *E. coli* ATCC 25922	This study

Str^s – Streptomycin sensitive, Str^r – Streptomycin resistance, Amp^r – Ampicillin resistance, Zeo^r– Zeocin resistance, Imp^r– Imipenem resistance, Cmp^r– Chloramphenicol resistance. *- truncation.

TABLE S3: Antibiotics and their sublethal concentrations used for excision assay in vitro.

**Antibiotics**	**NZ-1 (No IS*Aba125*)**	**N-SK9 (Single copy of IS*Aba125*)**	**N-SK10 (two copies of IS*Aba125*)**
Ciprofloxacin (CIP)	1.5ng/ml	1.5ng/ml	1.5ng/ml
Doxycycline hyclate (DOX)	20ng/ml	20ng/ml	20ng/ml
Gentamicin (GEN)	1.5µg/ml	1.5µg/ml	1.5µg/ml
Kanamycin (KAN)	600ng/ml	600ng/ml	600ng/ml
Mitomycin C (MMC)	10ng/ml	10ng/ml	10ng/ml
Neomycin (NEO)	20µg/ml	20µg/ml	20µg/ml
Rifampin (RIF)	7µg/ml	7µg/ml	7µg/ml
Tetracycline (TET)	50ng/ml	50ng/ml	50ng/ml
Trimethoprim (TMP)	60ng/ml	60ng/ml	60ng/ml

Table S4: Relevant bacterial strains used in this study.

Strains	Relevant genotype and/or phenotype	Source/ reference
V. cholerae		
N16961	WT clinical O1 El Tor strain, Str^r	Bangladesh, 1971 (24)
C6709	WT clinical O1 strain, with HapR function, Str^r	Peru, 1991 (24)
$HapR^+$ Str^s N16961	WT clinical O1 strain, with HapR function, Str^s	Lab stock
IDH06781	WT clinical non- O1/non- O139 strain, Str^r	Lab stock
C6709-Transformant	Transformed strain, with HapR function, Str^r, Amp^r, Zeo^r, Imp^r	This study
N16961 $HapR^+$-Transformant	Transformed strain, with HapR function, Str^s, Amp^r, Zeo^r, Imp^r	This study
Providencia stuartii MV493	WT, Clinical strain	Lab stock
Klebsiella Pneumoniae 14-B1	WT, Clinical strain	Lab stock
Pseudomonas aeruginosa AK1	WT, Clinical strain	Lab stock
E. coli		
β2163	F^- RP4-2-Tc: Mu Δ*dapA*: *erm-pir*	(25)
FCV14	DH5 λ*pir*+Δ*xerC*: *aph*	(25)
ATCC 25922	Reference strain, O6 serotype ETEC (O6:H1)	Lab stock
DH5α	*fhuA2Δ(argF-lacZ)U169 phoA glnV44 Φ80Δ(lacZ)M15 gyrA96 recA1 relA1 endA1 thi-1 hsdR17*	Lab stock

Str^s – Streptomycin sensitive, Str^r – Streptomycin resistance, Amp^r – Ampicillin resistance, Zeo^r– Zeocin resistance, Imp^r– Imipenem resistance.

TABLE S5: Antibiotics and their sublethal concentrations against *V. cholerae*, $HapR^+$ Str^s N16961 and Str^r C6709 strains used in chitin assay.

**Antibiotics**	***Vibrio cholerae* $HapR^+$ strains**	
	**C6709** (Str^r)	**N16961** (Str^s)
Ampicillin (AMP)	400ng/ml	400ng/ml
Ciprofloxacin (CIP)	1ng/ml	0.8ng/ml
Gentamicin (GEN)	4µg/ml	4µg/ml
Mitomycin C (MMC)	10ng/ml	8ng/ml
Rifampin (RIF)	3.5µg/ml	3.5µg/ml
Trimethoprim (TMP)	80ng/ml	80ng/ml

Str^s – Streptomycin sensitive, Str^r – Streptomycin resistance.

Legends

Fig S1: Heat map showing antibiotic susceptibility profiles of *V. cholerae* IDH06781, $HapR^+$ N16961, C6709 and the transformants of N16961 and C6709. *E. coli* ATCC 25922 was used as the reference strain. Antibiotic susceptibility was determined by disc diffusion assays and analyzed according to the CLSI guidelines. The disc concentration mentioned after the antibiotic name. Resistant (R; Red); Intermediate (I; Blue); Susceptible (S; Green). Figure was created using RStudio_Version.4.4.2.

Fig S2: Transformation efficiency of N16961 and C6709 in different antibiotic pressure. **(A)** Number of Transformants and the transformation efficiency of $HapR^+$ str^sN16961 strain **(B)** Number of Transformants and the transformation efficiency of $HapR^+$ str^s N16961 strain. Data was analyzed by unpaired t-test, values expressed as mean ± SEM of all the three experiments using the GraphPad Prism version.10.9. The names of the antibiotics are abbreviated as Ampicillin (AMP), Ciprofloxacin (CIP), Gentamicin (GEN), Mitomycin C (MMC), Rifampin (RIF), Trimethoprim (TMP).

Table S1: Oligonucleotides used in this study

Table S2: Relevant plasmids used in the study, transposons amplified and reporter strains constructed with their genotypes.

Table S3: Antibiotics and their sublethal concentrations used for excision assay in vitro.

Table S4: Relevant bacterial strains used in this study.

Table S5: Antibiotics and their sublethal concentrations against *V. cholerae*, $HapR^+$ str^s N16961 and str^r C6709 strains used in chitin assay.
