# Supplementary figures and images for: Investigating the molecular transmission dynamics of blaNDM in antibiotic-selective environments
Source: J Bacteriol. 2025 Aug 11;207(9):e00133-25. doi: 10.1128/jb.00133-25 (PMC12445084; doi:10.1128/jb.00133-25)

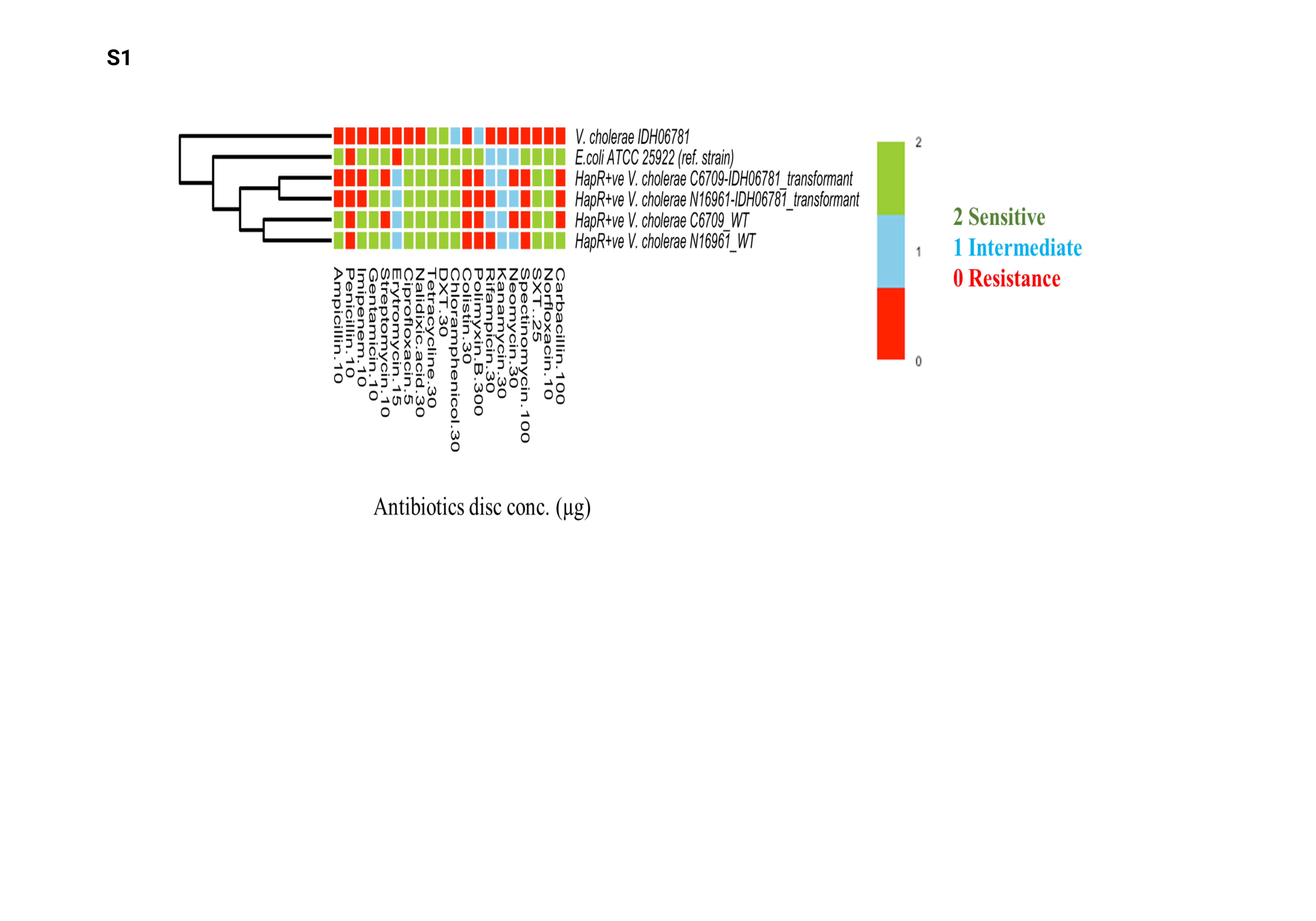

Supplement: Figure S1 — Heat map showing antibiotic susceptibility profiles of V. cholerae. [file jb.00133-25-s0002.tiff]

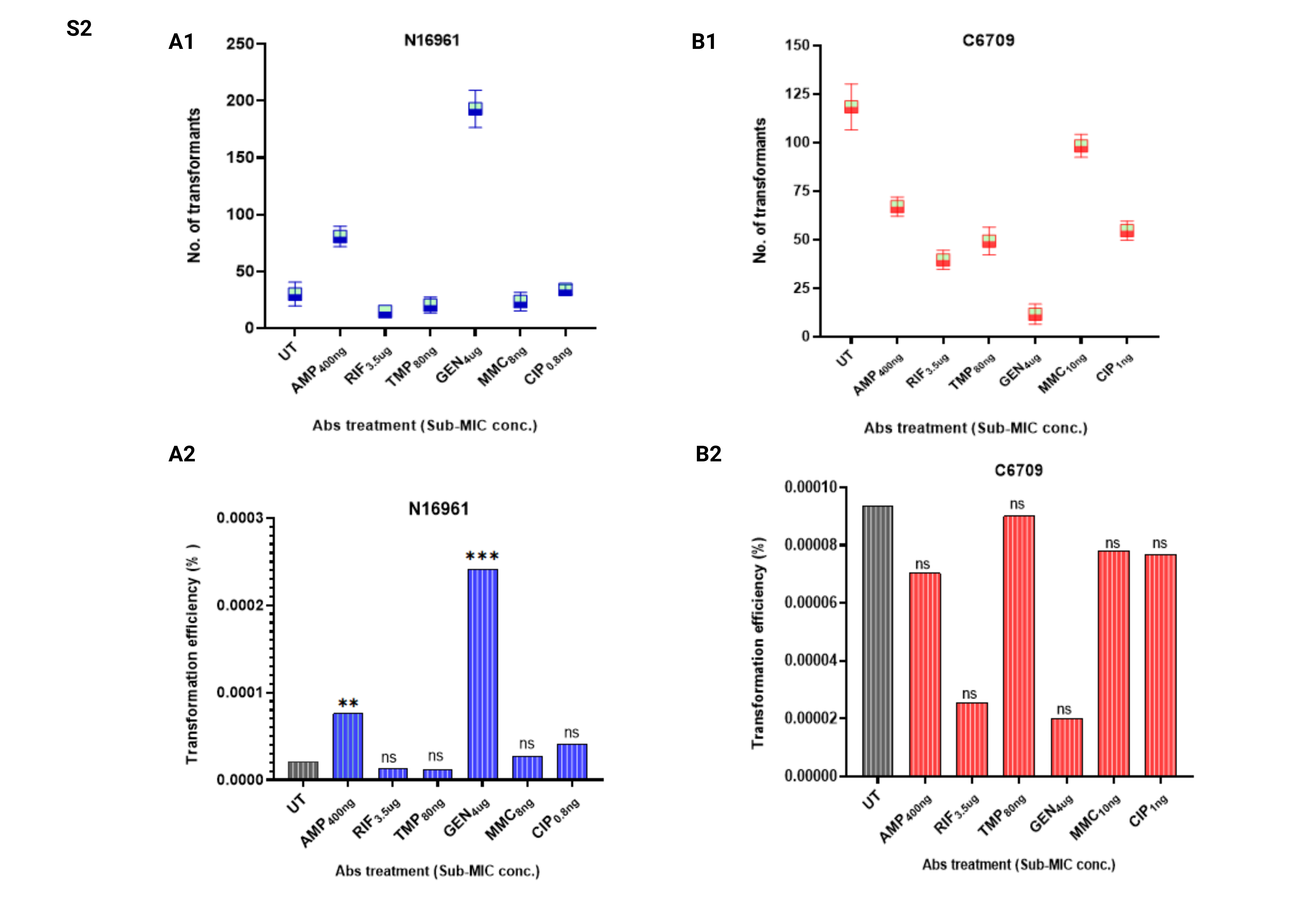

Supplement: Figure S2 — Transformation efficiency of N16961 and C6709 in different antibiotic pressures. [file jb.00133-25-s0003.tiff]
